# THE EFFECTS OF GRANDPARENTAL CARE ON MENTAL HEALTH AMONG CHINESE GRANDPARENTS: A LIFE COURSE PERSPECTIVE

**DOI:** 10.1093/geroni/igad104.3071

**Published:** 2023-12-21

**Authors:** Jiahui Lyu

**Affiliations:** University of Florida, Gainesville, Florida, United States

## Abstract

As the population continues to age, grandparental care has become a more prevalent form of childcare, particularly in China. Limited research has investigated the effects of grandparental care on grandparents’ mental health and the conclusions are ambiguous with both positive and negative outcomes being found. In this study, a life course perspective is adopted to examine the mechanisms of such effects. Using data from the China Health and Retirement Longitudinal Study (2011–2013), I conducted logistic regression models to examine the association between various stages of grandparental care and depressive symptoms among Chinese grandparents between the ages of 45 and 80 (N = 3946). Specifically, this study examines the impact of transitions into or out of grandparental care on depression and the potential moderating role of gender. Results show that grandparents who stop providing care for grandchildren are less likely to experience depression compared to those who provide no grandparental care (OR = 0.78). Additionally, grandmothers who provide ongoing grandparenting tend to have a higher risk of experiencing depression. The findings presented in this study offer valuable insights into the various mental health implications of grandparental care in the Chinese context, underscoring the significance of accounting for gender differences. This study also highlights the need for evidence-based policies and interventions that promote the well-being of older adults.

